# Effect of early detubularization on urethro-intestinal anastomosis during robot-assisted radical cystectomy and intracorporeal neobladder among bladder cancer patients

**DOI:** 10.3389/fonc.2025.1680595

**Published:** 2025-12-11

**Authors:** Jong Ho Park, Hakmin Lee, Sangchul Lee, Sung Kyu Hong, Seok-Soo Byun, Jong Jin Oh

**Affiliations:** 1 Department of Urology, Seoul National University Bundang Hospital, Seongnam, Republic of Korea; 2 Department of Urology, Seoul National University College of Medicine, Seoul, Republic of Korea

**Keywords:** bladder cancer, radical cystectomy, robotic surgical procedures, postoperative complications, anastomotic leak

## Abstract

**Purpose:**

Urethro-intestinal anastomosis (UIA) leak is a critical complication of robot-assisted radical cystectomy (RARC) with intracorporeal neobladder (ICN). Although early detubularization of ileal loop has been proposed to facilitate tension-free anastomosis, clinical evidence of its benefits in preventing UIA leaks is limited. We assessed its impact on UIA leak rates in bladder cancer patients undergoing RARC with ICN.

**Methods:**

We retrospectively identified 580 patients who underwent radical cystectomy at Seoul National University Bundang Hospital between 2003-2025, of which 147 met inclusion criteria for RARC with ICN and were analyzed. We retrospectively reviewed 580 patients who underwent radical cystetomy at Seoul National University Bundang Hospital between 2003-2025. Patients were grouped by detubularization timing: before (early) or after (conventional) UIA. Baseline and perioperative features were compared. Univariable analysis and multinomial logistic regression identified potential predictors of UIA leaks and evaluated the independent effect of early detubularization.

**Results:**

Among 147 eligible patients, 93 underwent early and 54 underwent conventional detubularization. UIA leaks occurred in 6.1% (9/147), lower in the early group (2.2% vs. 13.0%, P = 0.031). Early detubularization (P = 0.031), length of hospital stay (P = 0.001), and prior abdominal surgery (P = 0.165) were potential predictors using a liberal selection threshold (P < 0.20). Multinomial regression identified early detubularization as an independent protective factor (OR, 0.154; 95% CI, 0.030–0.784; P = 0.024). Model goodness-of-fit was significant (χ² = 9.775, df = 4, P = 0.044; Nagelkerke R² = 0.096).

**Conclusions:**

Early detubularization appears to reduce UIA leaks following RARC with ICN. Our findings support its adoption as a technical refinement to improve anastomotic outcomes, though further prospective validation is needed.

## Introduction

Compared to open surgery, the use of robot-assisted radical cystectomy (RARC) has steadily grown over the past decade in the surgical management of muscle-invasive bladder cancer. There is accumulating evidence that supports the oncological safety and perioperative feasibility of RARC (1, 2). As urinary diversion is an essential component of RARC, intracorporeal urinary diversion has gained increasing interest due to its potential perioperative advantages, though its broader adoption remains limited owing to the technical demands and complexity of the procedure (2–4). Among intracorporeal options, the orthotopic neobladder remains the most technically demanding form of diversion. First described laparoscopically by Gill et al. in 2002 and subsequently standardized in robotic settings with promising functional and oncological outcomes, it has demonstrated both feasibility and efficacy (5–7).

One of the most clinically significant complications of neobladder reconstruction is urethro-intestinal anastomosis (UIA) leak that adversely affect recovery, prolong catheter dependence, and potentially compromise long-term continence (8–11). Although overall urinary leak rates have been reported across various robotic series, the inconsistent reporting and heterogeneous surgical techniques have made it difficult to clearly define the specific incidence and modifiable risk factors of UIA leak (12–15).

In this context, Almassi et al. proposed a modification to neobladder reconstruction that involves early detubularization of the ileal segment—performed prior, rather than later, to UIA. This approach was intended to facilitate improved mobility and alignment of the ileal segment to enable tension-free anastomosis and to potentially reduce the risk of UIA leaks (16). However, a dedicated clinical study is needed to quantitatively evaluate the true impact of this technical adjustment on postoperative UIA leaks.

Therefore, we aimed to assess the clinical efficacy of early detubularization to reduce UIA leaks using real-world outcomes of its application in routine surgical practice at our institution.

## Materials and methods

### Study design

We retrospectively analyzed the medical records of 580 bladder cancer patients who underwent radical cystectomy at Seoul National University Bundang Hospital in South Korea between October 2003 and January 2025.

Inclusion criteria included bladder cancer patients who underwent RARC with intracorporeal neobladder (ICN). Selected individuals were divided into two groups based on whether detubularization was performed before UIA (early group) or after UIA (conventional group). We collected the following variables: age at surgery, sex, body mass index (BMI), past medical history (including prior abdominal surgery), American Society of Anesthesiologists (ASA) physical status, operative time, estimated blood loss, length of hospital stay, status of urinary leak, and functional outcomes (urinary incontinence and UIA stricture). Postoperative complications were recorded and graded according to the Clavien–Dindo classification. Major complications were defined as Clavien-Dindo grade >2 complications.

Urinary leak was identified based on postoperative findings of either radiological evidence of contrast extravasation on abdominopelvic computed tomography (CT) or elevated creatinine levels in the surgical drain fluid suggestive of urinary leak. Elevated drain creatinine was defined as a drain-to-serum creatinine ratio > 1.0 (17). Drain creatinine was routinely measured in all patients while the drain was in place. Radiological evaluation was performed selectively when urinary leakage was clinically suspected or during routine postoperative surveillance imaging. For analytical purposes, postoperative urinary leak was classified into three distinct categories: no leak, leak originating from the UIA, and leaks from other sites.

All research and related protocols used in this study complied with the principles of the Declaration of Helsinki. This study was approved by the institutional review board (IRB) from Seoul National University Bundang Hospital (IRB number: B-2309-851-107). Written informed consent was obtained from all patients.

### Surgical technique

A six-port technique was used with a midline: the 12-mm robotic camera port was placed 1–2 cm above the umbilicus. The Veress needle was used to analyze peritoneal access and insufflation. Three 8-mm robotic ports and one 12-mm assistant port were placed 8 cm apart, respectively, under direct vision at the level of the umbilicus. An additional 5 mm assistant port was placed 6–7 cm cephalad from the umbilicus between the right arm port and the camera port.

An incision was made in the peritoneum along the lateral umbilical ligament and carried inferiorly to the level of the vas deferens. After the intestine was retracted, the retroperitoneum was opened and the iliac arteries were identified. Posterior to the bladder, the retroperitoneum was divided longitudinally and both ureters were identified, dissected upward and downward, held by U-tape, cut near the bladder after Hem-O-Lock clamping of proximal ends, and ligated with 5–0 silk. The pathology of both the ureteral margins were assessed on the frozen sections. Bilateral pedicles and the posterior pedicle were divided step-by-step after ligation and the anterior endopelvic fascia was separated with blunt dissection. The space of Retzius was developed, and the dorsal vein complex was ligated and divided. The urethra was then clipped and transected. We assessed the pathology of the urethral margin on frozen sections. The specimen was then placed in an Endo Catch specimen pouch. We then completed bilateral pelvic lymphadenectomy.

The Endo GIA stapler was used to isolate a 45–50 cm segment of ileum, which was 15–20 cm proximal to the ileocecal valve. After reconstituting bowel continuity, the ileal segment was folded into a U configuration to form an ileal loop. The distal 30 cm of ileum was used to create the urinary reservoir, which left a 15-cm proximal chimney for ureteral anastomosis. The apex of the ileal loop that would reach the urethral stump was identified to create the UIA.

**Figure 1:**
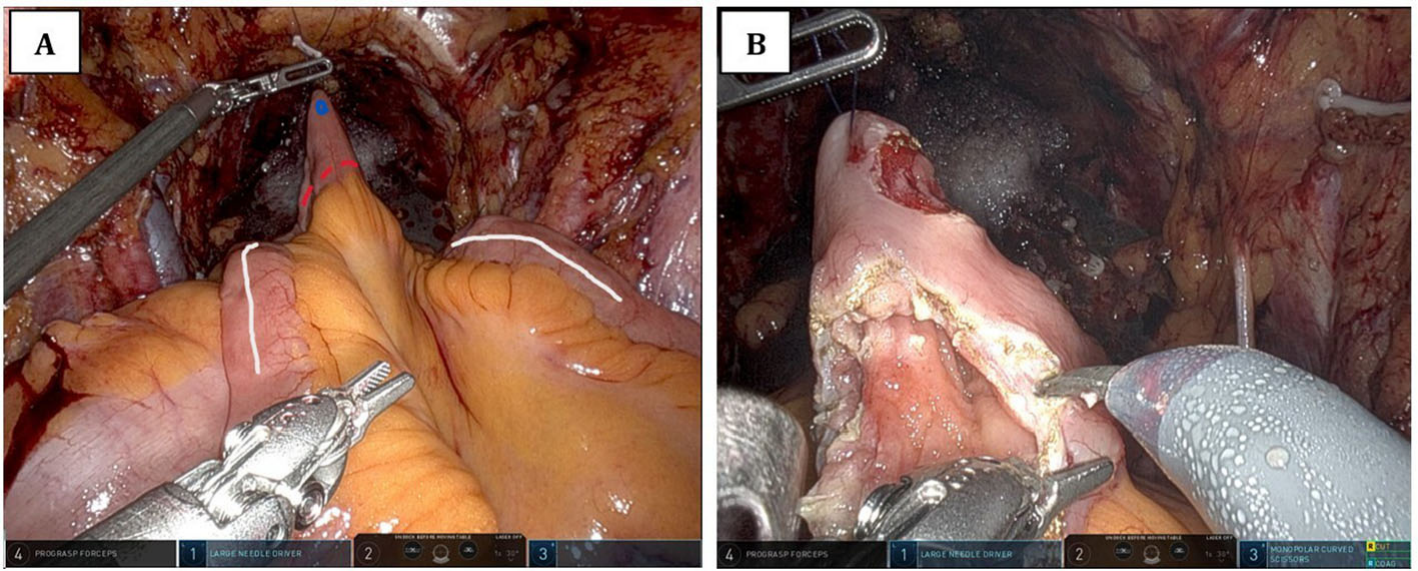
Intraoperative view of early detubularization of the ileal loop prior to urethro-intestinal anastomosis. **(A)** The schematic plan for early detubularization of the ileal loop. The white line indicates the antimesenteric border of the ileal loop. The blue dot marks the site of the urethro-intestinal anastomotic opening, which is created prior to detubularization. The dotted red line shows the planned incision for detubularization. **(B)** The surgical field after the anastomotic opening has been created and detubularization has been completed.

A hole was cut out in this dependent portion of ileum for UIA, and we performed early detubularization of the portion of the ileal loop near the anastomotic site (**Figure 1**). The UIA was performed with a 3–0 V-Loc suture, following which, a 22-French urethral catheter was placed. The steps of early detubularization and the UIA are demonstrated in **Supplementary Video 1**. The leak test was performed to test the integrity of the UIA with consecutive instillations of 50 to 100 ml normal saline through the urethral catheters. After completion of the UIA, the remnant ileal loop was detubularized along its antimesenteric border and subsequently folded and sutured along its long axis using a running 3–0 V-Loc suture to construct the urinary reservoir. The ureters were tailored to reach the ileal chimney and anastomosed in an end-to-side fashion to the ileal chimney. Just before completing each ureteral anastomosis, a single-J ureteral stent was inserted, which was brought out through the anterior neobladder wall and secured with an absorbable suture. The robot was undocked and the specimen extracted through an extension incision of the camera port. One Jackson-Pratt drain was placed, and the incisions were closed layer by layer.

### Statistical analyses

Baseline characteristics were compared using either an independent t-test or a Wilcoxon rank-sum test for continuous variables. Either a Chi-square test or Fisher’s exact test was utilized to analyze categorical variables. Continuous variables were described as mean with standard deviation (SD) for parametrically-distributed variables and median values with interquartile range (IQR) for nonparametric variables. Categorical variables were expressed by the frequency (%) of events. A Shapiro-Wilk test was used to perform a normality test for continuous variables.

Univariable analyses were conducted to identify potential predictors of UIA leak. Categorical variables were assessed with the same tests as those for baseline characteristics. Continuous variables were compared using one-way ANOVA or the Kruskal-Wallis test, depending on the distribution. *Post-hoc* analysis was not performed as the primary purpose was variable screening prior to multivariable modeling. Among the variables examined in the univariable analyses, those that met a liberal selection threshold (p < 0.20) and were considered clinically relevant were selected for inclusion in the multivariable model. To evaluate the independent effect of early detubularization and other covariates on the leak subtype, a multinomial logistic regression model was employed using “no leak” as the control. As a sensitivity analysis, we additionally constructed a multinomial logistic regression model including hospital length of stay to examine the potential influence of this post-treatment variable on leak outcomes. Furthermore, a separate binary logistic regression analysis was conducted for exploratory purposes, as is later discussed. The model included both categorical and continuous variables. Odds ratios (OR) with 95% confidence intervals (CI) were reported. Descriptive multicollinearity was evaluated among candidate variables using linear regression–based variance inflation factor (VIF) analysis. Model fitness was evaluated using the likelihood ratio test and Nagelkerke’s pseudo-R². A *post hoc* power analysis was performed to estimate the statistical power of detecting differences in UIA leak rates between the two groups.

All the statistical analyses were performed using IBM SPSS Statistics ver. 22.0 (IBM Co., Armonk, NY, USA) and R version 4.3.2 (R Foundation for Statistical Computing, Vienna, Austria). A p-value of less than 0.05 was considered statistically significant.

## Results

### Baseline characteristics

Among 580 patients, a total of 147 patients met the inclusion and the exclusion criteria for our study. Of these, 54 patients were included in the conventional group and 93 patients were included in the early group. **Table 1** demonstrates the clinical and demographic characteristics of the two groups.

**Table 1:** Baseline characteristics of the study population.

Variables	Conventional (N = 54)	Early (N = 93)	P value
Age (year)	61.0 (51.8-67.0)	64.0 (59.0-71.0)	0.010
BMI (kg/m²)	24.9 (3.7)	24.9 (2.6)	0.959
Sex			0.505
Men	49 (90.7)	81 (87.1)	
Women	5 (9.3)	12 (12.9)	
HTN			0.182
No	34 (63.0)	48 (51.6)	
Yes	20 (37.0)	45 (48.4)	
DM			0.200
No	44 (81.5)	67 (72.0)	
Yes	10 (18.5)	26 (28.0)	
ASA classification			< 0.001
1	23 (42.6)	11 (11.8)	
2	29 (53.7)	61 (65.6)	
> 3	2 (3.7)	21 (22.6)	
Baseline serum creatinine (mg/dl)	0.92 (0.83-1.08)	0.96 (0.84-1.08)	0.357
Baseline serum eGFR (ml/min)	84.0 (70.0-95.6)	79.5 (67.9-90.8)	0.125
Prior abdominal surgery			0.455
No	42 (77.8)	77 (82.8)	
Yes	12 (22.2)	16 (17.2)	

Data are presented as n (%) or mean (SD) or median values (IQR). N, number of patients; BMI, body mass index; HTN, Hypertension; DM, Diabetes mellitus; ASA, American Society of Anesthesiologists; eGFR, estimated glomerular filtration rate; SD, standard deviation; IQR, interquartile range.

### Perioperative outcomes

**Table 2** summarizes perioperative outcomes between the early and the conventional groups. Surgical procedure time, estimated blood loss, and length of hospital stay were all significantly lower in the early group than in the conventional group (all p < 0.001). The incidence of postoperative urinary leak also differed significantly between the two groups (P = 0.031). UIA leak was observed in 13% of the conventional group and 2.2% of the early group, with an overall incidence of 6.1% (9/147). Other types of leaks occurred at similar rates in both groups.

**Table 2:** Perioperative outcomes.

Variables	Conventional (N = 54)	Early (N = 93)	P value
Surgical procedure time (min)	505 (429-576)	345 (308-425)	< 0.001
Estimated blood loss (ml)	675 (500-1000)	300 (200-450)	< 0.001
Length of hospital stay (d)	24 (14-32)	13 (12-16)	< 0.001
Complications within 90 days			0.070
Non	9 (16.7)	30 (32.3)	
Minor	17 (31.5)	18 (19.4)	
Major	28 (51.9)	45 (48.4)	
Postoperative urinary leakage			0.031
No leak	42 (77.8)	81 (87.1)	
UIA leak	7 (13.0)	2 (2.2)	
Other types of leaks	5 (9.3)	10 (10.8)	
UIA stricture	7 (7.5)	1 (1.9)	0.259
Urinary incontinence	10 (10.8)	6 (11.1)	0.946
90-day readmission	17 (31.5)	28 (30.1)	0.862
90-day mortality	0 (0)	1 (1.1)	> 0.999

Data are presented as n (%) or mean (SD) or median values (IQR). N, number of patients; UIA, Urethro, intestinal anastomosis; SD, standard deviation; IQR, interquartile range.

There were no statistically significant differences between groups in rates of UIA stricture (p = 0.259) or urinary incontinence (p = 0.946), although the early group showed numerically fewer strictures. There was no statistically significant difference in the 90-day complication rates (p = 0.070), although the early group tended to have a high rate of non-complicated cases. Readmission rates were comparable (p = 0.862), and there was one case of 90-day mortality in the early group due to postoperative septic shock.

### Univariable analyses for leak subtypes

Univariable analyses were performed to identify factors associated with the three categories of postoperative urinary leak: no leak, UIA leak, and other types of leaks (**Table 3**). Among the tested variables, early detubularization (p = 0.031), length of hospital stay (p = 0.001), and prior abdominal surgery (p = 0.165) were identified as potential predictors based on a liberal selection threshold of p < 0.20.

**Table 3:** Univariable analysis of factors associated with postoperative urinary leak subtypes.

Variables	No leak (N = 123)	UIA leak (N = 9)	Others (N = 15)	P value
Age (year)	62.0 (57.0-68.0)	67.0 (55.5-71.5)	65.0 (58.0-73.0)	0.485
BMI (kg/m²)	24.9 (3.2)	25.8 (3.2)	24.5 (2.3)	0.624
Sex				0.424
Men	110 (90.7)	7 (77.8)	13 (87.7)	
Women	13 (10.6)	2 (22.2)	2 (13.3)	
HTN				0.307
No	72 (58.5)	4 (44.4)	6 (40.0)	
Yes	51 (41.5)	5 (55.6)	9 (60.0)	
DM				0.684
No	91 (74.0)	8 (88.9)	12 (80.0)	
Yes	32 (26.0)	1 (11.1)	3 (20.0)	
ASA classification				0.943
1	27 (22.0)	3 (33.3)	4 (26.7)	
2	76 (61.8)	5 (55.6)	9 (60.0)	
> 3	20 (16.3)	1 (11.1)	2 (13.3)	
Baseline serum creatinine (mg/dl)	0.93 (0.83-1.09)	0.99 (0.78-1.17)	0.93 (0.82-1.05)	0.685
Baseline serum eGFR (ml/min)	81.0 (17.6)	76.6 (12.2)	81.8 (15.0)	0.737
Prior abdominal surgery				0.165
No	101 (82.1)	5 (55.6)	13 (86.7)	
Yes	22 (17.9)	4 (44.4)	2 (13.3)	
Surgical procedure time (min)	405 (330-480)	405 (338-588)	375 (320-555)	0.732
Estimated blood loss (ml)	400 (200-650)	600 (350-900)	300 (200-600)	0.261
Length of hospital stay (d)	14 (12-20)	30 (13-40)	20 (15-37)	0.001
Early detubularization				0.031
No	42 (34.1)	7 (77.8)	5 (33.3)	
Yes	81 (65.9)	2 (22.2)	10 (66.7)	

P-values were calculated by comparing the three groups (no leak, UIA leak, and other types of leaks) using one-way ANOVA or the Kruskal-Wallis test for continuous variables, and chi-square or Fisher’s exact test for categorical variables, as appropriate.

Data are presented as n (%) or mean (SD) or median values (IQR). N, number of patients; UIA, Urethro, intestinal anastomosis; Others – other types of leaks; BMI, body mass index; HTN, Hypertension; DM, Diabetes mellitus; ASA, American Society of Anesthesiologists; eGFR, estimated glomerular filtration rate; SD, standard deviation; IQR, Interquartile range.

### Multinomial logistic regression analysis

A multinomial logistic regression model was then constructed to evaluate the independent effects of early detubularization and prior abdominal surgery on leak subtypes, using “no leak” as the control (**Table 4**). Although statistically associated with leak subtypes in the univariable analysis, the length of hospital stay was excluded from the multivariable model as it was considered a post-treatment variable that could be influenced by the outcome (i.e., leak) and pose a risk of reverse causality. A sensitivity model including this variable is provided in **Supplementary Table 1**. Prior to model estimation, multicollinearity was assessed using VIF analysis, and no severe multicollinearity was detected among the included variables (all VIFs < 5).

**Table 4:** Multinomial logistic regression for predictors of UIA leaks and other types of leaks.

UIA leak							
Variables		B	Std. Error	Wald	df	OR (95% CI)	P value
Prior abdominal surgery	No	–	–	–	–	1 [Reference]	–
	Yes	1.239	0.736	2.836	1	3.452 (0.816-14.596)	0.092
Early detubularization	No	–	–	–	–	1 [Reference]	–
	Yes	-1.873	0.831	5.075	1	0.154 (0.030-0.784)	0.024
Other types of leaks							
Variables		B	Sth. Error	Wald	df	OR (95% CI)	P value
Prior abdominal surgery	No	–	–	–	–	1 [Reference]	–
	Yes	- 0.347	0.795	0.190	1	0.707 (0.149-3.361)	0.663
Early detubularization	No	–	–	–	–	1 [Reference]	–
	Yes	0.030	0.580	0.003	1	1.030 (0.330-3.213)	0.959

Model fit statistics: Final model χ² = 9.775, df = 4, P = 0.044; Nagelkerke R² = 0.096.

The reference outcome category for the multinomial logistic regression model was "no leak".

UIA, Urethro, intestinal anastomosis; B, regression coefficient; Std. Error, standard error; Wald, Wald chi, square test; df, degrees of freedom; OR, odds ratio; CI, confidence interval.

In the final model, early detubularization was significantly associated with a reduced risk of UIA leak compared to the conventional group in the multinomial regression analysis (OR: 0.154, 95% CI: 0.030–0.784, P = 0.024). Early detubularization did not demonstrate any significant associations with predicting other types of leaks in the multinomial logistic regression analysis (OR: 1.030, 95% CI: 0.330–3.213, P = 0.959). Although a trend toward increased UIA leak was observed (OR: 3.452, 95% CI: 0.816–14.596), prior abdominal surgery was not a statistically significant predictor of either UIA leak (P = 0.092) or other leaks (P = 0.663). The overall model fit was supported by the likelihood ratio test (χ² = 9.775, df = 4, P = 0.044). Nagelkerke R² for the final model was 0.096, suggesting modest explanatory power.

## Discussion

Our study assessed the impact of early detubularization on postoperative urinary leaks in patients undergoing RARC with ICN. Our results demonstrate that early detubularization is independently associated with a significantly low risk of UIA leak (OR: 0.154, 95% CI: 0.030–0.784, P = 0.024). This association remained significant in a multinomial logistic regression model when adjusted for other variables. No factor was significantly associated with non-UIA leaks.

The UIA is widely recognized as one of the most critical and vulnerable components of urinary reconstruction, with leak rates reported to range from 2% to 15% depending on surgical experience, technique, and study population (18–22). Although various factors, such as ischemia, tension, and tissue handling have been implicated in anastomotic integrity, specific modifiable intraoperative techniques to reduce UIA leaks have rarely been examined systematically.

Our findings suggest that the sequence of reconstruction plays a pivotal role. Performing detubularization before urethral anastomosis may allow the detubularized ileal segment to reach the urethra easily and reduce the tension at the anastomotic site. This interpretation is supported by Almassi et al. (16), who proposed early detubularization to overcome challenges in ICN and emphasized that it facilitates an anatomically favorable, tension-free approximation of tissues. This aligns with our clinical observation that early detubularization facilitates a technically smooth anastomosis and may thereby contribute to low risk of leaks. However, our study did not include direct intraoperative measurements of bowel mobility or anastomotic tension. As objective quantification methods for these parameters remain limited in current practice, this represents an important area for future methodological development.

Although baseline characteristics, such as ASA classification and age, showed differences between early and conventional groups, these factors did not demonstrate a statistically significant association with leaks in univariable analysis. Hence, this reduces the likelihood that they serve as confounders. Prior abdominal surgery was also included in the analysis based on general surgical considerations as it has been associated with increased technical complexity due to adhesions in pelvic procedures (13). However, the lack of significant association with UIA leaks, in either univariable or multivariable analysis, suggested abdominal surgery plays a limited role as an independent predictor in this context.

We also note that although the length of hospital stay is associated with leaks in univariable analysis, this factor was excluded from the multivariable model due to its status as a post-treatment variable that could bias the model through reverse causality (23). To address this, we present a supplementary sensitivity model including hospital stay in **Supplementary Table 1**. The final multivariable model demonstrated acceptable goodness-of-fit (χ² = 9.775, p = 0.044), although the explanatory power was modest (Nagelkerke R² = 0.096). This reflects the limited number of events but remains consistent with the direction of the observed effect.

Our study has several limitations that should be considered. First, the retrospective nature and single-center design may limit the generalizability of the findings and increase its susceptibility to selection bias. Second, the relatively small number of UIA leak events (n = 9) may reduce the statistical power and the stability of the multivariable regression estimates, irrespective of the observed significance.

Although the multinomial regression model reached statistical significance (χ² = 9.775, P = 0.044), the Nagelkerke R² value of 0.096 indicates modest explanatory power. Additionally, the events-per-variable ratio was below the commonly recommended threshold of 10 (24), raising concern for potential model overfitting. While we applied a liberal variable selection criterion (P < 0.20) and confirmed the absence of multicollinearity via VIF analysis, these steps cannot fully address the limitations inherent to low event counts.

Furthermore, due to the retrospective nature of the study, an *a priori* sample size calculation was not feasible. Instead, we conducted a *post hoc* power analysis based on the observed group sizes (54 *vs*. 93) and UIA leak rates (13.0% *vs*. 2.2%), which yielded a power of approximately 73% at a two-sided alpha level of 0.05. Although this falls short of the conventional 80% threshold typically recommended for clinical research (25), it still suggests a moderate capacity to detect a true effect under the given study conditions. As such, while our findings are statistically significant, they should be interpreted with appropriate caution.

Third, there is a likelihood of temporal bias, as patients in the conventional detubularization group were more likely to have undergone surgery during the earlier phase of our institution’s experience. This reflects the fact that early detubularization was introduced later as part of procedural refinements. The observed differences in perioperative outcomes—such as operative time, estimated blood loss, and length of hospital stay—were likely influenced not only by the technical modification but also by the surgeon’s learning curve, which progressed over time.

Given the possibility that the experience of the surgeon and the institutional learning curve could confound the observed associations between early detubularization and UIA leaks, we explored the feasibility of applying propensity score matching to reduce temporal and selection bias. However, given the limited number of events, especially in the UIA leak group (n = 9), matched sample sizes became too small to draw meaningful statistical inferences. As a result, we decided to retain the full cohort and instead applied an exploratory temporal adjustment using the variable ‘days-since-diagnosis of first-case’.

Although univariable analysis of ‘days-since-diagnosis of first-case’ showed no significant association with leak subtype (P = 0.201), an inspection of rank distributions revealed that the “no leaks” and “other types of leak” groups shared similar central tendencies, while the “UIA leak” group differed substantially (**Supplementary Table 2**). Therefore, to more precisely assess whether early detubularization retained its significance independent of the time when surgical intervention was performed, we refined the leak outcome into “UIA leak” versus “all others” and performed a supplementary binary logistic regression analysis including ‘days-since-diagnosis of the first case’ (**Supplementary Table 3**). In this model, ‘days-since-diagnosis of the first case’ was included in the initial step of the stepwise logistic regression but was subsequently removed due to lack of statistical significance (P = 0.754). This variable was not retained in the final model shown in **Supplementary Table 3**. In contrast, early detubularization remained independently associated with a low risk of UIA leak (OR: 0.153; 95% CI: 0.030–0.778; P = 0.024). The univariable analysis performed to identify candidate factors for multivariate modeling is summarized in **Supplementary Table 4**.

Although this method—using ‘days-since-diagnosis of first-case’ as a variable—serves as an alternative to more robust temporal stratification methods such as propensity score matching, this suggests that the observed benefit of early detubularization is unlikely to be explained solely by accumulated surgical procedure experience.

Overall, to validate the independent effects of early detubularization, it will be essential to conduct further studies with prospectively-balanced cohorts, standardized surgical protocols, and appropriate temporal adjustments. In addition, future studies should incorporate leak severity indicators—such as the need for reoperation and time to resolution—as well as broader functional outcomes, to more comprehensively evaluate the protective effect of early detubularization.

## Conclusion

Early detubularization was independently associated with a significantly reduced risk of UIA leak following RARC with ICN. This effect likely stems from improved anastomotic geometry and reduced tension, as previously described in the literature of surgical techniques. These findings support the consideration of early detubularization as a modifiable surgical procedure step to enhance reconstructive outcomes. Future prospective, multi-institutional, randomized studies are warranted to validate this strategy and to assess the long-term functional outcomes.

## Data Availability

The datasets generated during and/or analyzed during the current study are available from the corresponding author on reasonable request.
