# Supplementary material for: Effect of early detubularization on urethro-intestinal anastomosis during robot-assisted radical cystectomy and intracorporeal neobladder among bladder cancer patients
Source: Front Oncol. 2025 Dec 11;15:1680595. doi: 10.3389/fonc.2025.1680595 (PMC12739548; doi:10.3389/fonc.2025.1680595)
Supplement: Supplementary file 1 [file Table1.docx]

Supplementary Table 1. Sensitivity analysis: Multinomial logistic regression including length of hospital stay for predictors of UIA leaks and other types of leaks

| UIA leak | | | | | | | |
| --- | --- | --- | --- | --- | --- | --- | --- |
| Variables | | B | Std. Error | Wald | df | OR (95% CI) | P value |
| Length of hospital stay | | 0.083 | 0.033 | 6.360 | 1 | 1.087 (1.019-1.160) | 0.012 |
| Prior abdominal surgery | No | - | - | - | - | 1 [Reference] | - |
|  | Yes | 1.145 | 0.774 | 2.189 | 1 | 3.143 (0.690-14.324) | 0.139 |
| Early detubularization | No | - | - | - | - | 1 [Reference] | - |
|  | Yes | -0.905 | 0.949 | 0.911 | 1 | 0.404 (0.063-2.596) | 0.340 |
| Other types of leaks | | | | | | | |
| Variables | | B | Std. Error | Wald | df | OR (95% CI) | P value |
| Length of hospital stay | | 0.109 | 0.030 | 13.057 | 1 | 1.115 (1.051-1.183) | < 0.001 |
| Prior abdominal surgery | No | - | - | - | - | 1 [Reference] | - |
|  | Yes | -0.172 | 0.828 | 0.043 | 1 | 0. 842 (0.166-4.266) | 0.835 |
| Early detubularization | No | - | - | - | - | 1 [Reference] | - |
|  | Yes | 1.395 | 0.792 | 3.105 | 1 | 4.037 (0.855-19.062) | 0.078 |

Model fit statistics: Final model χ² = 30.280, df = 6, P < 0.001; Nagelkerke R² = 0.278.
The reference outcome category for the multinomial logistic regression model was "no leak".
UIA - Urethro-intestinal anastomosis; B - regression coefficient; Std. Error - standard error; Wald - Wald chi-square test; df - degrees of freedom; OR - odds ratio; CI - confidence interval.

Supplementary Table 2. Distribution of surgery dates (days since diagnosis of first case) across urinary leak subtypes

| Variable | No leak (N = 123) | UIA leak (N = 9) | Others (N = 15) | P value |
| --- | --- | --- | --- | --- |
| Days since diagnosis of first case (d) | 5917 (5133-6610) | 5098 (1494-6239) | 6239 (5105-6785) | 0.201 |

Kruskal-Wallis test was used to compare the “days since diagnosis of first case” among the three leak subgroups. P-value reflects overall group comparison. The mean ranks were 74.16 for the no leak group, 53.11 for the UIA leak group, and 85.20 for the others group, with a P value of 0.201. Data are presented as median values (IQR – interquartile range). N - number of patients; UIA - Urethro-intestinal anastomosis; Others – other types of leaks.Supplementary Table 3. Binary logistic regression analysis for predictors of UIA leak

| Variables | | B | Std. Error | Wald | df | OR (95% CI) | P value |
| --- | --- | --- | --- | --- | --- | --- | --- |
| Prior abdominal surgery | No | - | - | - | - | 1 [Reference] | - |
|  | Yes | 1.273 | 0.732 | 3.019 | 1 | 3.570 (0.850-14.997) | 0.082 |
| Early detubularization | No | - | - | - | - | 1 [Reference] | - |
|  | Yes | -1.876 | 0.829 | 5.117 | 1 | 0.153 (0.030-0.778) | 0.024 |

The binary logistic regression model was constructed using a likelihood ratio backward stepwise method (entry threshold = 0.05, removal = 0.10). Initial input variables were days-since-diagnosis of the first case, early detubularization, prior abdominal surgery, and EBL. Days-since-diagnosis and EBL were removed during stepwise selection (P = 0.754 and 0.447, respectively) and are not shown in the final model. The final model demonstrated acceptable goodness-of-fit (Hosmer–Lemeshow test: P = 0.961), and explained variance was modest (Nagelkerke R² = 0.171).
The dependent binary variable was categorized as “UIA leak” vs. “all others”. The reference category for the binary logistic regression model was “all others”.
UIA - Urethro-intestinal anastomosis; B - regression coefficient; Std. Error - standard error; Wald - Wald chi-square test; df - degrees of freedom; OR - odds ratio; CI - confidence interval; EBL – estimated blood loss.

Supplementary Table 4. Univariable analysis of factors associated with either UIA leak or all other outcomes

| Variables | All others (N = 138) | UIA leak (N = 9) | P value |
| --- | --- | --- | --- |
| Age (year) | 63.0 (57.0-68.5) | 67.0 (55.5-71.5) | 0.856 |
| BMI (kg/m²) | 24.9 (3.1) | 25.8 (3.2) | 0.384 |
| Sex |  |  | 0.279 |
| Men | 123 (89.1) | 7 (77.8) |  |
| Women | 15 (10.9) | 2 (22.2) |  |
| HTN |  |  | 0.509 |
| No | 78 (56.5) | 4 (44.4) |  |
| Yes | 60 (43.5) | 5 (55.6) |  |
| DM |  |  | 0.454 |
| No | 103 (74.6) | 8 (88.9) |  |
| Yes | 35 (25.4) | 1 (11.1) |  |
| ASA classification |  |  | 0.793 |
| 1 | 31 (22.5) | 3 (33.3) |  |
| 2 | 85 (61.6) | 5 (55.6) |  |
| > 3 | 22 (15.9) | 1 (11.1) |  |
| Baseline serum creatinine (mg/dl) | 0.93 (0.83-1.08) | 0.99 (0.78-1.17) | 0.500 |
| Baseline serum eGFR (ml/min) | 81.1 (17.3) | 76.6 (12.2) | 0.446 |
| Prior abdominal surgery |  |  | 0.067 |
| No | 114 (82.6) | 5 (55.6) |  |
| Yes | 24 (17.4) | 4 (44.4) |  |
| Surgical procedure time (min) | 405 (330-480) | 405 (338-588) | 0.443 |
| Estimated blood loss (ml) | 375 (200-613) | 600 (350-900) | 0.168 |
| Length of hospital stay (d) | 14 (13-21) | 30 (13-40) | 0.079 |
| Early detubularization |  |  | 0.012 |
| No | 47 (34.1) | 7 (77.8) |  |
| Yes | 91 (65.9) | 2 (22.2) |  |
| Days since diagnosis of first case (d) | 5945 (5131-6661) | 5098 (1494-6239) | 0.129 |

Statistical comparisons were made between patients with UIA leak and those with all other outcomes to identify variables for inclusion in the binary logistic regression model.
Data are presented as n (%) or mean (SD) or median values (IQR). BMI - body mass index; HTN - Hypertension; DM - Diabetes mellitus; ASA - American Society of Anesthesiologists; eGFR - estimated glomerular filtration rate; UIA - Urethro-intestinal anastomosis; SD - standard deviation; IQR - Interquartile range.
